# Younger age and previous exposure to radiation therapy are correlated with the severity of chemotherapy-induced thrombocytopenia

**DOI:** 10.3332/ecancer.2019.906

**Published:** 2019-02-26

**Authors:** Sergo Mkhitaryan, Samvel Danielyan, Lilit Sargsyan, Lusine Hakobyan, Samvel Iskanyan, Samvel Bardakchyan, Ruzanna Papyan, Jemma Arakelyan, Karmen Sahakyan, Tatevik Avagyan, Armen Tananyan, Armen Muradyan, Gevorg Tamamyan

**Affiliations:** 1 Department of Oncology, Yerevan State Medical University, Yerevan 0025, Armenia; 2 Clinic of Chemotherapy, Muratsan Hospital Complex, Yerevan State Medial University, Yerevan 0025, Armenia; 3 Armenian Pediatric Hematology and Oncology Group, Yerevan 0014, Armenia; 4 Hematology Centre, Yerevan 0014, Armenia; 5 Department of Histology,Yerevan State Medical University, Yerevan 0025, Armenia; 6 National Oncology Centre, Yerevan 0052, Armenia; 7 Department of Urology, Yerevan State Medical University, Yerevan 0025, Armenia; 8 Advanced Oncology Program, University of Ulm, 89081 Ulm, Germany

**Keywords:** chemotherapy-induced thrombocytopenia, platelet transfusion, clinically significant bleeding, radiation therapy

## Abstract

**Background:**

Chemotherapy-induced thrombocytopenia (CIT) is a significant complication of cancer therapy. Data on the optimal management approaches of this morbidity in children and young adults are still limited.

**Aim:**

The aim of the study is to estimate the frequency and severity of CIT and associated clinically significant bleeding in children and young adults with solid tumours and haematologic malignancies.

**Methods:**

For this retrospective, hospital-based study, children (0–18 y) and young adults (19–40 y) with different types of solid tumours and haematologic malignancies who received chemotherapy at the Muratsan Hospital Complex of Yerevan State Medical University were identified from the patients’ database and included in the study (overall 122 patients). Thrombocytopenia was defined as a decrease of platelet count below <100 × 10^9^/L. For assessing bleeding, WHO scale had been used.

**Results:**

Overall, the whole group of patients received 430 chemotherapy cycles. During 131 (31.6%) chemotherapy cycles, patients developed CIT. The study revealed a statistically significant inversely proportional correlation between the age and the severity of CIT. Another important finding of the study was that the patients, who previously were exposed to radiation therapy, were more likely to develop CIT, than those who have not received radiation therapy (68% and 28.7%, *p* = 0.001). From 430 cycles of chemotherapy, 31 (7.2%) cycles reported to have bleeding events.

**Conclusion:**

Our study showed that clinically significant thrombocytopenia and bleeding are quite rare among children and young adults. Younger age and previous exposure to radiation therapy are positively correlated with the severity of thrombocytopenia. Larger studies are needed to investigate these findings.

## Introduction

Thrombocytopenia is a frequent complication of cancer therapy, but there is still no consensus among physicians regarding the management of this important problem [1–3]. As a result of chemotherapy-induced thrombocytopenia (CIT), various types of bleeding can be observed: from petechiae and ecchymoses to life-threatening bleeding. Among other consequences of CIT are chemotherapy dose reductions and delays, which decrease the efficacy of delivered therapy. The most widely used method to treat CIT associated bleeding is the platelet transfusion [1, 4]. However, from the clinicians’ perspective, not all bleeding has a significant clinical impact or requires transfusion [5].

Current recommendations either favour prophylactic platelet transfusions, i.e. when the platelet count drops below 10 × 10^9^/L (in some centres lower or higher thresholds are implemented) or on-demand transfusions, meaning when bleeding occurs [6–9]. Data on the optimal management of CIT in paediatric patients are very limited and the recommendations are mostly based on the results from adults’ studies [1, 5].

## Materials and methods

This retrospective study was conducted at the Clinic of Chemotherapy of Muratsan Hospital Complex of Yerevan State Medical University (Armenia). We reviewed the charts of the patients with solid tumours and haematologic malignancies aged 0–40 years, who were treated at the clinic between 1 January 2008 and 31 December 2015. Patients from 0 to 18 years old formed the cohort of children (54% of entire group), and from 19 to 40 years were considered as young adults. Those patients, who were admitted to the clinic to receive non-cytotoxic treatment were excluded from the study cohort. The final study cohort included 122 patients, who received 430 chemotherapy courses in total. 67 (55%) patients were males. The median age for the entire group was 18 y (28 y for solid tumours and 17 y for haematologic malignancies). 42 (34.4%) patients were in the age group of 0–10 years, 32 (26.2%) patients 11–20 y, 21 (17.2%) patients in 21–30 y group and 27 (22.2%) patients were in the age group of 31–40 years. 91 (75%) patients had a blood cancer and 31 (25%) patients presented with solid tumours. Out of 66 patients in the group of 18 years and younger 14 had solid tumours (21.2%), and in older age group out of 56 patients 17 had solid tumours (30.4%).

Thrombocytopenia was considered a decrease of platelet count in peripheral blood below 100 × 10^9^/ L, and National Cancer Institute (NCI), USA scale was incorporated for grading: grade 1—75–100 × 10^9^/L, grade 2—50–74 × 10^9^/ L, grade 3—25–49 × 10^9^/L and grade 4—below 25 × 10^9^/L [5]. Grade 3 and grade 4 thrombocytopenia were considered as a clinically significant thrombocytopenia. To assess the severity of bleeding, we used the WHO bleeding scale: 0 = no bleeding, 1 = petechia, 2 = mild blood loss, 3 = severe blood loss and 4 = debilitating blood loss [10].

Descriptive statistical methods were incorporated for the statistical analysis of the collected data. For comparing continuous variables ANOVA-univariate, and for categorical variables, chi-square tests were used. Analyses were performed using IBM SPSS Statistics 20.

## Results

At the admission for the entire study group, the median platelet count was 221 × 10^9^/L. At diagnosis, only 9% of patients with haematologic malignancies had grade 4 thrombocytopenia and 4% had grade 3 thrombocytopenia. In the solid tumour group, none of the patients were found to have thrombocytopenia at diagnosis.

The information about chemotherapy was available for 414 (96.3%) out of 430 courses. Thrombocytopenia had been documented during 131 (31.6%) courses; in particular, CIT occurred during chemotherapy courses for acute lymphoblastic leukaemia (ALL) in 54% (42/77), 38.1% (16/42) of Ewing sarcoma chemotherapy courses, 92.3% of germ cell tumours (12/13), 4.8% in Hodgkin lymphoma (8/165) and 42.5% of non-Hodgkin lymphoma (31/73). During chemotherapy cycles for the patients with acute myeloid leukaemia (AML) (five courses), chronic myelomonocytic leukaemia [1], neuroblastoma [4] and osteosarcoma [9] thrombocytopenia were reported in every cycle. In our cohort of patients, the majority of thrombocytopenia cases occurred during the treatment of haematologic malignancies, than in solid tumours (during 87 cycles for haematologic malignancies and 44 cycles for solid tumours). However, if we take the actual rates of thrombocytopenia, it was more common in solid tumours (47%; 44 out of 93 cycles) than in haematologic malignancies (27%; 87 out of 321).

The exact number of platelets was available for 405 (94.2%) courses. Grade 4 thrombocytopenia was documented during 39 courses (9.6%), grade 3 thrombocytopenia during 28 courses (6.9%), grade 2 and grade 1, accordingly in 35 (8.6%) and 29 (7.1%) of courses.

In our cohort, grade 4 thrombocytopenia was more common among males (11.48%), than in females (7.653%), but the statistical analysis could not show significant correlation between patient sex and severity of thrombocytopenia (*p* = 0.274).

The study revealed that statistically significant correlation between patients’ age and the incidence of thrombocytopenia (*p* < 0.001), as well as age and severity of thrombocytopenia (*p* < 0.001), i.e. with ageing the incidence and severity of thrombocytopenia was decreasing. Clinically significant thrombocytopenia (grade 3 and grade 4) were more commonly documented in 0–10 and 11–20 age groups than in older age groups: 57.1% of grade 3 and 64.1% of grade 4 thrombocytopenia occurred in 0–10 age group, and 28.6% of grade 3 and 25.6% of grade 4 thrombocytopenia in 11–20 age group. Similar results were also documented in 0–18 and 18–40 age groups, i.e. 78.6% of grade 3 and 89.7% of grade 4 thrombocytopenia occurred in the 0–18 age group (*p* < 0.001).

**Figure 1.**
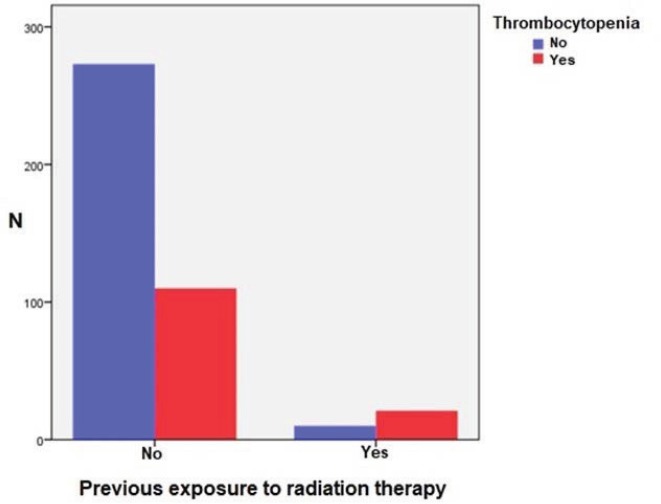
CIT and previous exposure to radiation therapy.

In the analysis, the study revealed statistically significant correlation between the incidence and severity of thrombocytopenia and previous exposure to radiation therapy (*p* = 0.001). 21 (68%) out of 31 patients, who previously received radiation therapy, developed CIT. In the group of patients (383 chemotherapy cycles), who didn’t receive radiation therapy previously, CIT was much rarer—28.7% (110 chemotherapy cycles) (Figure 1). In the radiation therapy group, clinically significant thrombocytopenia, i.e. grades 3 and 4, were more common, respectively, 13.3% and 20%, than in non-radiation group, respectively, 6.4% and 8.8% (*p* < 0.001). This means that previous exposure to radiation therapy is a risk factor for developing clinically significant CIT.

Regarding previous exposure to chemotherapy, no statistically significant correlation was noted (*p* = 0.193) between the number of previously received chemotherapy courses and the incidence and severity of resulted thrombocytopenia.

## Bleeding

During the entire study period, only 7.2% (31/430) courses presented with bleeding. The vast majority of bleeding episodes were documented during the treatment of haematologic malignancies: during 83% of chemotherapy courses for AML patients bleed (five out of six cycles) and 17.5% for ALL (14/80). In solid tumours group, the majority of bleeding episodes were during osteosarcoma treatment (44%; during four cycles out of nine).

67% of haemorrhages were WHO grade 1, 23% grade 2, 7% were grade 3 and 3% grade 4. The average duration of bleeding was 5.6 days. In the entire cohort, there was only one episode of intracranial bleeding; the outcome was lethal and the patient was an 18 years old female with AML (platelet count was 17,490/L).

During the study, ten cases of bleeding were recorded when the absolute number of platelets was <10 × 10^9^/L; seven episodes when the platelets were in the range of 11–20 × 10^9^/L; three cases in the range of 21–30 × 10^9^/L and three cases in the range of 31–50 × 10^9^/L; five cases in the range of 51–75 × 10^9^/L and one case when the platelet number was >100 – 10^9^/L. Clinically significant bleeding was documented in two cases when the platelet count was <10 × 10^9^/L and in one case when the platelet count was in the range of 11–20 × 10^9^/L. With higher platelet numbers, no clinically significant bleeding was noted.

## Platelet transfusion

In the entire study cohort, platelet transfusions were performed only during 30 (7%) chemotherapy courses. In average, four small packages of platelets (1–10) were used per person. No post-transfusion complications were recorded with platelet transfusions.

## Discussion

Thrombocytopenia is an important and frequent complication of chemotherapy (in our study, thrombocytopenia was noted in 1/3 of cases), and many questions still remain open about its management. The aim of our study was to estimate the frequency and features of CIT and associated clinically significant bleeding in children and young adults. As it was shown, at admission of patients with haematologic malignancies (mostly AML and ALL), only 13% had clinically significant thrombocytopenia; in solid tumour patients, none presented with thrombocytopenia on admission.

From the clinical point of view, the importance of thrombocytopenia is in associated bleeding. It was previously reported that bleeding occurs more often in haematologic malignancies than in solid tumours, which is explained not only by direct exposure to chemotherapy but also by associated haematopoietic system damage caused by haematologic malignancies [11–13]. In our study, although the most of thrombocytopenia cases occurred during the treatment of haematologic malignancies, thrombocytopenia was more common in solid tumours (47% versus 27%, respectively). This could be explained by the fact that more than half of chemotherapy cycles in haematologic cohort were Hodgkin lymphoma cases (165/321), in which CIT is very rare—8%, but in ALL and non-Hodgkin lymphoma, the rate of CIT was, respectively, 54% and 42.5%.

The study showed that the frequency of thrombocytopenia and bleeding were in the youngest groups, and with ageing, the incidence was decreasing.

An important finding of the study was that in addition to the relationship between patient’s age and severity of thrombocytopenia, previous exposure to radiation therapy was a risk factor for clinically significant thrombocytopenia.

Although it is noted that there is no absolutely safe amount of platelets to prevent bleeding [1, 5], our study has shown that in children and young adults, the risk of bleeding increases when the number of platelets decreases to the mark of 10,000/L. However, clinically significant bleeding, needing interventions, is very rare (in our cohort it was <1%). In our study group, although the number of platelet transfusions was low, no transfusion-related complication was noted.

## Conclusion

Our study showed that clinically significant thrombocytopenia and bleeding are quite rare among children and young adults. Younger age and previous exposure to radiation therapy are positively correlated with the severity of thrombocytopenia. Further larger and prospective studies are needed to examine our findings, as well as the approaches for prevention and treatment of CIT.

## Funding statement

This research work has been completely self-financed by the authors.

## Conflicts of interest

The authors declare that they have no conflicts of interest.
